# OCT4 Silencing Triggers Its Epigenetic Repression and Impairs the Osteogenic and Adipogenic Differentiation of Mesenchymal Stromal Cells

**DOI:** 10.3390/ijms20133268

**Published:** 2019-07-03

**Authors:** Ricardo Malvicini, Diego Santa-Cruz, Natalia Pacienza, Gustavo Yannarelli

**Affiliations:** Laboratorio de Regulación Génica y Células Madre, Instituto de Medicina Traslacional, Trasplante y Bioingeniería (IMeTTyB), Universidad Favaloro-CONICET, Solís 453, Buenos Aires 1078, Argentina

**Keywords:** mesenchymal stem cells, OCT4, multipotency, differentiation, epigenetics

## Abstract

Mechanisms mediating mesenchymal stromal/stem cells’ (MSCs) multipotency are unclear. Although the expression of the pluripotency factor OCT4 has been detected in MSCs, whether it has a functional role in adult stem cells is still controversial. We hypothesized that a physiological expression level of OCT4 is important to regulate MSCs’ multipotency and trigger differentiation in response to environmental signals. Here, we specifically suppressed OCT4 in MSCs by using siRNA technology before directed differentiation. OCT4 expression levels were reduced by 82% in siOCT4-MSCs, compared with controls. Interestingly, siOCT4-MSCs also presented a hypermethylated OCT4 promoter. OCT4 silencing significantly impaired the ability of MSCs to differentiate into osteoblasts. Histologic and macroscopic analysis showed a lower degree of mineralization in siOCT4-MSCs than in controls. Moreover, OCT4 silencing prevented the up-regulation of osteoblast lineage-associated genes during differentiation. Similarly, OCT4 silencing resulted in decreased MSC differentiation potential towards the adipogenic lineage. The accumulation of lipids was reduced 3.0-fold in siOCT4-MSCs, compared with controls. The up-regulation of genes engaged in the early stages of adipogenesis was also suppressed in siOCT4-MSCs. Our findings provide evidence of a functional role for OCT4 in MSCs and indicate that a basal expression of this transcription factor is essential for their multipotent capacity.

## 1. Introduction

The octamer-binding transcriptional factor 4 (OCT4) plays a key role in maintaining the self-renewal and pluripotency of embryonic stem cells (ESCs) [1]. OCT4 also positively regulates the expression of other transcription factors that form the core pluripotency network, including sex-determining region Y-box 2 (SOX2) and NANOG [2]. Accordingly, OCT4 is the most significant factor that induces the reprogramming of somatic cells into ESC-like induced pluripotent stem cells (iPSCs), as without OCT4, no reprogramming occurs [3,4]. Loss of OCT4 expression is associated with decreased self-renewal and differentiation of ESCs into trophectoderm. On the contrary, overexpression of OCT4 triggers the differentiation of ESCs into primitive endoderm and mesoderm [5]. Thus, a balanced level of OCT4 is required to maintain the self-renewal of undifferentiated ESCs, indicating that this transcription factor does not function in a binary on–off manner. Recent evidence suggests that OCT4 is involved in ESCs’ early cell fate determination as well [6]. Whether OCT4 expression plays an important role for the self-renewal and differentiation potential of adult stem cells, however, is still controversial.

Mesenchymal stromal/stem cells (MSCs) are adult stem cells that can differentiate into multiple nonhematopoietic cell lineages, including osteoblasts, chondrocytes, adipocytes, and myotubes [7]. It is well known that MSCs comprise a heterogeneous population of cells differing in morphology, rates of proliferation, and differentiation potential. A subpopulation of small, spindle-shaped cells is obtained in low-density cultures of MSCs at early passages. This subpopulation can be selected by serum deprivation and presents long telomeres and enhanced expression of OCT4 and other characteristic genes of early progenitor cells [8]. In addition, we previously found that MSCs obtained from human umbilical cord (UC-MSCs) exhibit a higher frequency of CD146+ stromal progenitors and an enhanced OCT4 expression and telomerase activity, compared with bone marrow (BM)-derived MSCs [9]. Considering their neonatal tissue origin, these characteristics are reasonable and result in an improved plasticity and better regenerative properties [9,10]. Nonetheless, OCT4 promoter methylation is similar in UC- and BM-MSCs (50%), and significantly higher than in ESCs (less than 5%) [9]. This latter observation infers that OCT4 expression may be epigenetically restricted in MSCs and suggests a possible mechanism for the limited plasticity of these cells when compared with ESCs. In agreement with this notion, the ectopic expression of OCT4 can enhance the adipogenic and ostegenic differentiation of MSCs [11]. Moreover, some reports have shown that OCT4 overexpression improves the proliferation and colony formation of MSCs, suggesting that the expression of this factor maintains the stemness of MSCs [12,13]. Experiments using ectopic OCT4 expression, however, usually increase its level more than 100-fold and may not reflect the behavior of MSCs under physiological conditions. In this context, previous reports have found that the expression of OCT4 only shows an increase up to five-fold after stimulation of MSCs with differentiation signals, a response that involves a de-differentiation step before differentiation [14,15]. On the contrary, a study by Tsai et al. showed decreased differentiation potential into osteogenic, adipogenic, and chondrogenic lineages in MSCs with OCT4 knockdown, although possible mechanisms mediating this effect were not investigated [13]. Based on these data, we hypothesized that a physiological expression level of OCT4 is important to regulate MSCs’ multipotency and trigger differentiation in response to differentiation stimuli. Thus, in the present study, we investigated whether OCT4 silencing impairs osteogenic and adipogenic differentiation of MSCs by blocking the response of early lineage commitment regulators. We employed a DNA vector-based RNA-mediated interference approach to suppress OCT4 gene expression in MSCs. We found that the specific silencing of OCT4 leads to its epigenetic repression by hypermethylation of the OCT4 promoter. Moreover, OCT4 silencing prevents the up-regulation of the transcription factors runt-related transcription factor 2 (RUNX2) and peroxisome proliferator-activated receptor gamma (PPARγ2), which are critical regulators for the commitment of MSCs into ostegenic and adipogenic lineages, respectively. Our findings provide evidence of a functional role for OCT4 in MSCs and suggest a mechanism that may be important to regulate the interaction of these cells with the microenvironment.

## 2. Results

### 2.1. BM-MSC Characterization and OCT4 Silencing

**Figure 1:**
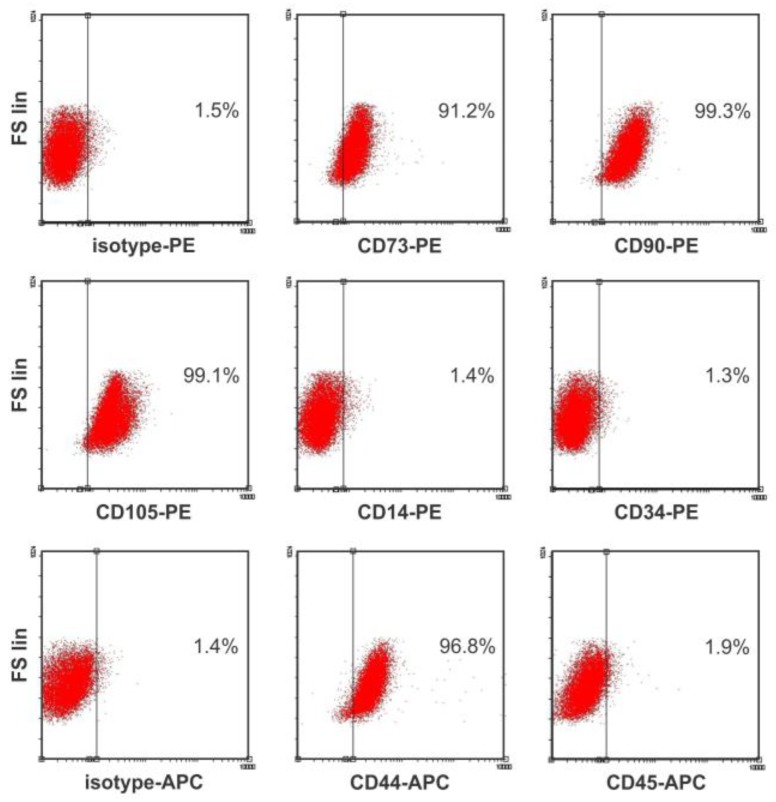
Phenotypic characterization of bone marrow-derived mesenchymal stromal/stem cells (BM-MSCs). Surface marker expression levels in BM-MSCs were analyzed by flow cytometry. MSCs highly express (>90%) the stromal determinants CD44, CD73, CD90, and CD105, and are negative (<2%) for the expression of hematopoietic markers (CD14, CD34, CD45). Representative dot-plots of three independent assays.

**Figure 2:**
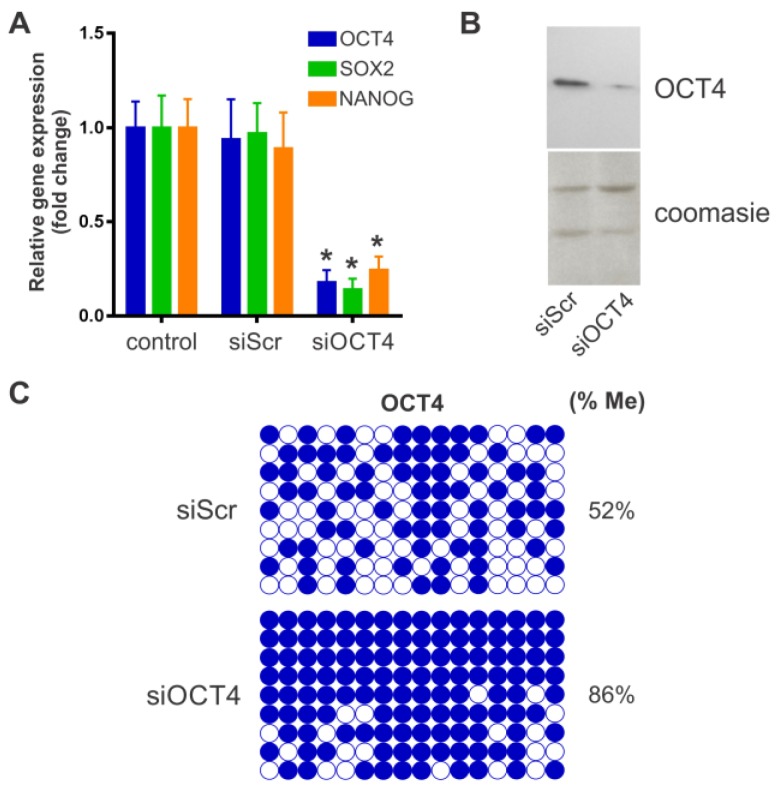
Downregulation of octamer-binding transcriptional factor 4 (OCT4) expression after RNA interference using OCT4 siRNA. BM-MSCs were incubated for 24 h with OCT4 siRNA (siOCT4) or a scrambled control siRNA (siScr) and transfected cells were then selected using puromycin. (**A**) Quantitative RT-PCR assay for expression of OCT4, SOX2, and NANOG in untreated MSCs (control), OCT4 siRNA silenced (siOCT4), and scrambled siRNA MSCs (siScr). Results were normalized against GAPDH as a reference gene and plotted relative to the expression level of untreated MSCs. Results are expressed as mean ± SD (*n* = 4 per group); *p < 0.001 derived from one-way ANOVA and Tukey’s multiple comparisons test. (**B**) Immunoblot with nuclear cell extracts from siOCT4 and siScr MSCs, using antibodies against OCT4. Total protein loading was determined by Coomasie blue staining. (**C**) Bisulfite sequencing analysis of OCT4 promoter in siOCT4 and siScr MSCs. Open and closed circles indicate unmethylated and methylated CpGs, respectively. The overall percentage of methylation is noted to the right of each panel.

Phenotypic characterization of BM-MSCs was carried out using flow cytometry analysis of cell surface markers. A positive immunostaining was consistently obtained for the stromal markers CD44, CD73, CD90, and CD105 without the expression of the hematopoietic markers CD14, CD34, and CD45 (Figure 1). In addition, BM-MSCs showed multilineage differentiation potential. Thus, BM-MSCs used in this study conformed with the minimal criteria defining MSCs [16]. Down-regulation of OCT4 expression in BM-MSCs was achieved by using a plasmid vector to produce a specific hairpin siRNA against this transcription factor (siOCT4). MSCs transfected with the siOCT4 plasmid showed a reduction of 82% in OCT4 expression, compared with untreated MSCs (Figure 2A). The use of a scrambled siRNA negative control (siScr) showed no significant effect (Figure 2A). Of note, the expression of the pluripotency factors SOX2 and NANOG also decreased in OCT4-silenced MSCs, a result consistent with the role of OCT4 as an essential regulator of pluripotency. Nuclear OCT4 expression was detected in siScr-MSCs by Western Blot, whereas siOCT4-MSCs showed a significant reduction in nuclear OCT4 content (Figure 2B). Epigenetic mechanisms are important for the regulation of OCT4 expression and pluripotency. In this sense, we next studied whether the silencing of OCT4 was associated with a change in the methylation status of the OCT4 promoter. Interestingly, we found that siOCT4-MSCs presented a hypermethylated OCT4 promoter in comparison with siScr-MSCs (86% vs. 52%, respectively; *p* < 0.0001 between groups derived from unpaired *t*-test) (Figure 2C). These data indicate that the down-regulation of OCT4 in MSCs is able to trigger epigenetic modifications that further repress OCT4 expression.

### 2.2. OCT4 Silencing Impairs MSC Osteogenic Differentiation

**Figure 3:**
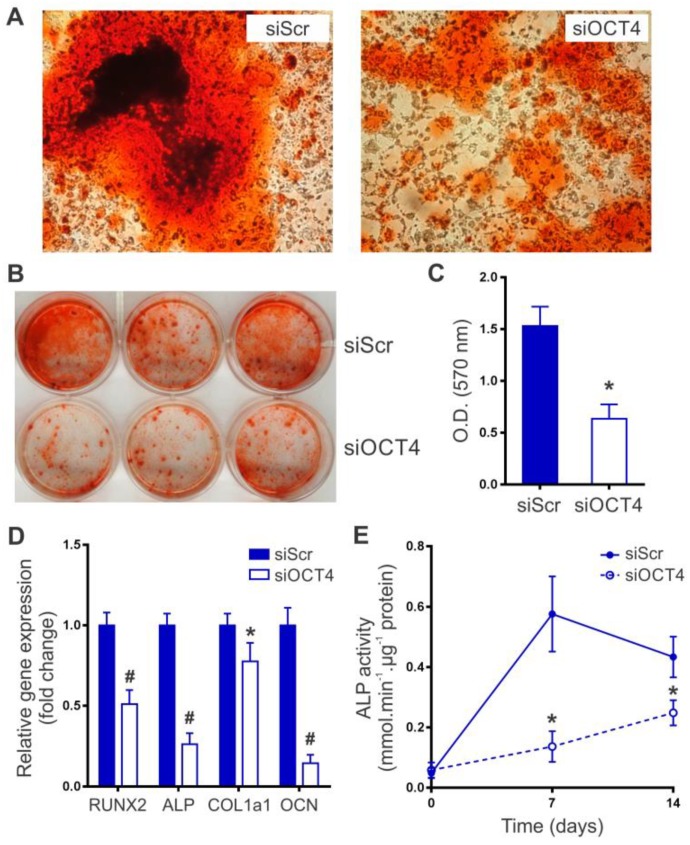
OCT4 silencing impairs MSC osteogenic differentiation. OCT4 siRNA (siOCT4) and scrambled siRNA (siScr) MSCs were cultured in osteogenic differentiation medium. (**A**) Representative images of Alizarin Red S staining to visualize mineralized bone matrix at day 21 (magnification x200). (**B**) Representative macroscopic image of wells after MSC osteogenic differentiation. (**C**) Quantification of calcium mineral content as determined by absorbance at 570 nm after dye elution from wells in (**B**). Results are expressed as mean ± SD (*n* = 6 per group); *p < 0.0001 derived from unpaired t test. (**D**) Quantitative RT-PCR analysis of osteogenic markers at seven days after differentiation. Results are expressed as mean ± SD (*n* = 4 per group); *p < 0.05 and #*p* < 0.001 between groups derived from unpaired *t*-test. (**E**) Kinetics of alkaline phosphatase (ALP) activity during osteogenic differentiation. Results are expressed as mean ± SD (*n* = 6 per group); *p < 0.001 between groups derived from unpaired *t*-test.

MSCs were incubated in osteogenic induction media to investigate the effect of OCT4 silencing on osteoblast lineage differentiation. We first determined the presence of calcium mineralization by using Alizarin Red S staining after 21 days of differentiation. Histologic and macroscopic analysis showed a significantly lower degree of mineralization in siOCT4-MSCs than in siScr-MSCs (Figure 3A,B). A quantitative analysis of the calcified mineral demonstrated a 2.4-fold decrease in the ability of siOCT4-MSCs to produce mineralization (Figure 3C). We next studied whether OCT4 silencing affects the expression of osteoblast lineage-associated genes during differentiation. Runt-related transcription factor 2 (RUNX2) is a critical regulator for the commitment of MSCs to the osteoblast lineage and modulates the expression of several bone matrix-related genes, such as alkaline phosphatase (ALP), type I collagen (COL1a1), and osteocalcin (OCN) [17]. After seven days of differentiation, the expression of RUNX2 was significantly lower in siOCT4-MSCs in comparison with siScr-MSCs (Figure 3D). Consequently, siOCT4-MSCs showed a clear repression of ALP, COL1a1, and OCN (Figure 3D). The major difference was observed in the expression of OCN (86% reduction in siOCT4-MSCs compared with siScr-MSCs), a late differentiation marker expressed in mature osteoblasts. In siScr-MSCs, ALP activity first increased at day 7 and then started to decrease as the cells matured (Figure 3E), a behavior that meets the well-established pattern of osteoblast differentiation [18]. Interestingly, we found that OCT4 silencing prevented or delayed the increase of ALP activity during differentiation (Figure 3E). Overall, these data indicate that OCT4 expression is required for an optimum osteogenic differentiation of MSCs.

### 2.3. OCT4 Silencing Prevents MSC Adipocyte Differentiation

**Figure 4:**
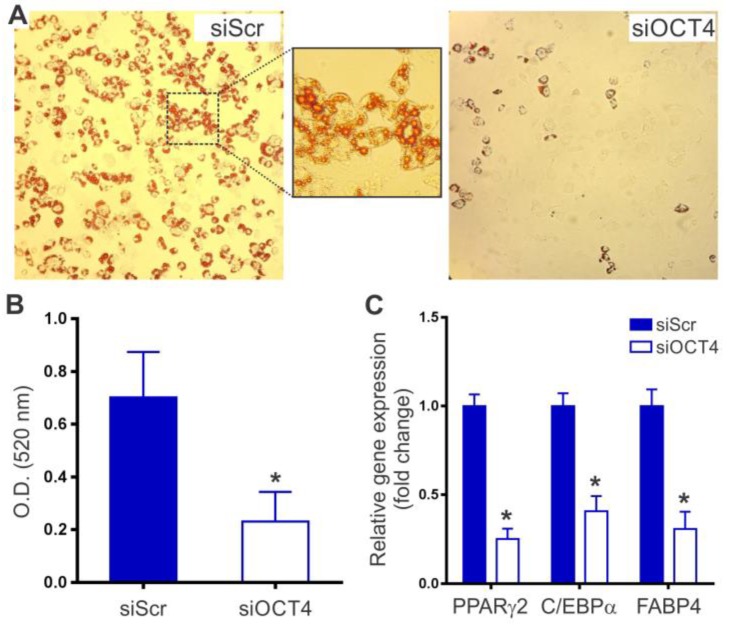
OCT4 silencing prevents MSC adipocyte differentiation. OCT4 siRNA (siOCT4) and scrambled siRNA (siScr) MSCs were tested for their ability to differentiate into adipogenic lineages by culture in differentiation medium for 15 days. (**A**) Representative images of Oil Red O staining to visualize lipid accumulation after 15 days of differentiation (magnification ×200). (**B**) Lipid accumulation was quantified by dye extraction and spectrophotometry at 520 nm. Results are expressed as mean ± SD (*n* = 6 per group); *p < 0.001 derived from unpaired *t*-test. (**C**) Quantitative RT-PCR analysis of adipogenic markers at seven days after differentiation. Results are expressed as mean ± SD (*n* = 4 per group); *p < 0.0001 between groups derived from unpaired *t*-test.

Finally, we asked whether OCT4 silencing also impairs the capacity of MSCs to differentiate into adipocytes. After 15 days of culture in adipogenic induction medium, siScr-MSCs acquired a marked adipogenic phenotype, characterized by the accumulation of lipid-rich vacuoles. The presence of lipid droplets was further confirmed by positive Oil Red O staining (Figure 4A). In contrast, only a small percentage of siOCT4-MSCs (less than 30%) were able to differentiate into adipocytes. It has been demonstrated that the degree of Oil Red O staining is proportional to the extent of adipocyte differentiation [19]. Quantitation of lipid accumulation by Oil Red O extraction showed a 3.0-fold decrease in the conversion of siOCT4-MSCs into adipocytes when compared with siScr-MSCs (Figure 4B). Moreover, the up-regulation of transcription factors engaged in the early stages of adipogenesis, such as peroxisome proliferator-activated receptor gamma (PPARγ2) and CCAAT enhancer binding protein alpha (C/EBPα), was suppressed by more than 2.0-fold in siOCT4-MSCs (Figure 4C). It is well-known that PPARγ and C/EBPα activate de novo or enhance the expression of genes that characterize the adipocyte phenotype [20]. In agreement with this notion, the expression of fatty acid binding protein 4 (FABP4), the most abundantly expressed gene in mature adipocytes, significantly decreased in siOCT4-MSCs, compared with siScr-MSCs (Figure 4C). Thus, these data demonstrate that OCT4 expression is also required for the differentiation of MSCs into the adipogenic lineage.

## 3. Discussion

Mechanisms mediating MSC multipotency remain elusive. Although previous studies found that ectopic OCT4 expression increases proliferation, stemness, and differentiation potential of MSCs into osteoblasts, adipocytes, and chondrocytes [11,12], whether the physiological expression of this transcription factor regulates MSC multipotency is still unclear. Experiments forcing the expression of OCT4 in MSCs usually provide a more than 100-fold increase in OCT4 mRNA levels [11,21]. In these cases, the ectopic OCT4 expression drives a de-differentiation process, characterized by a significant genomic reorganization resembling genetic reprogramming to pluripotency. These changes maintain MSCs in an undifferentiated state and are associated with an increase in their differentiation potential [11,12]. This mechanism, however, may be different to what normally occurs when MSCs interact with their microenvironment. In this regard, it has been shown that the stem cell niche can determine the fate of stem cells [22]. We hypothesized that a certain basal expression level of OCT4 is required for the response of MSCs to different differentiation cues. Thus, in the present study, we specifically suppressed OCT4 expression in MSCs by using siRNA technology before directed differentiation. We found that the DNA methylation level of the OCT4 promoter significantly increased, indicating that the initial down-regulation of OCT4 further triggers its repression by epigenetic modifications. Of note, it has been shown that DNA methylation is the major epigenetic mechanism regulating the expression of stemness genes [23]. We also observed that the expression of other transcription factors of the pluripotency network, such as SOX2 and NANOG, significantly decreased in OCT4-silenced MSCs. The interaction between OCT4, SOX2, and NANOG has been extensively investigated in ESCs. OCT4 knockdown in ESCs leads to the down-regulation of the OCT4 and SOX2 promoter activities [24]. In addition, it has been demonstrated that NANOG is a downstream target of OCT4 and SOX2 [25]. Thus, our results suggest that similar regulatory mechanisms may operate in MSCs, with OCT4 as the main regulator of the expression of pluripotency genes. Most importantly, our data demonstrate that the suppression of OCT4 results in a decreased ability of MSCs to differentiate into osteogenic and adipogenic lineages, and hence, in a significant loss of multipotency. Previous studies have shown that SOX2 or NANOG over-expression can improve MSC differentiation into the osteogenic and chondrogenic lineages [12,26]. In contrast to OCT4 over-expression, NANOG over-expression has been shown to impair adipogenesis [12]. Therefore, the basal expression level of the pluripotency factor OCT4 may play a critical role in preserving the stemness and differentiation potential of MSCs by modulating the expression of SOX2 and NANOG. In fact, enhanced OCT4 transcriptional activity can initiate genetic reprogramming of somatic cells as it can later induce the up-regulation of endogenous SOX2 and NANOG to proceed with the reprogramming program [27].

It is well known that the characteristics and function of MSCs differ, depending on their tissue origin [28,29]. Interestingly, MSCs obtained from neonatal sources have demonstrated higher OCT4 expression and enhanced differentiation potential, compared to their adult counterparts [9,30]. Whereas MSCs can differentiate into osteoblast, adipocytes, and chondrocytes, only MSCs derived from neonatal tissues have shown an improved capacity to transdifferentiate towards other lineages, such as cardiomyocytes and neuronal cells [31,32,33,34]. In this regard, Ramkisoensing et al. demonstrated that human MSCs from fetal but not adult origin differentiate toward three major cardiovascular lineages, including cardiomyocytes, smooth muscle cells, and endothelial cells [31]. Moreover, UC-MSCs showed a greater extent of cardiomyocyte differentiation in vitro and provided a better short-term left ventricular function in an acute myocardial infarction model when compared with BM-MSCs [10]. We recently demonstrated that OCT4 expression is required for this transdifferentiation of MSCs into cardiomyocytes, a process that involves de-differentiation-mediated reprogramming [14]. Similarly, MSCs obtained from human placenta are able to transdifferentiate into dopaminergic neurons and can attenuate motor defects associated with Parkinson’s Disease [32]. All these data suggest that differences in basal OCT4 expression levels among MSCs obtained from different sources may account for the diverse plasticity shown by these cells. We previously found that epigenetic mechanisms, such as DNA methylation, can restrict OCT4 expression in MSCs [9]. In fact, the OCT4 promoter is about 50% methylated both, in UC- and BM-derived MSCs, whereas it is hypomethylated in ESC (<5%) and hypermethylated in terminally differentiated cells (i.e., >80% in fibroblasts) [9,35]. This mechanism may explain the significantly lower OCT4 expression, differentiation capacity, and the very low risk of tumor formation (if any) of MSCs compared with ESCs. Moreover, the epigenetic repression of the OCT4 promoter may also account for the limited difference in OCT4 expression levels between MSCs from neonatal vs. adult tissues (usually around two to five times higher in the former). This difference, which may be due to post-translational modifications [36], may suffice to justify the improved plasticity of neonatal tissue-derived MSCs. In this regard, differentiation processes involving a physiological de-differentiation step of MSCs are usually associated with an up-regulation of up to five times in OCT4 levels and a net gain in differentiation potential [14,15].

In conclusion, we found that suppressing the expression of the transcription factor OCT4 significantly impairs the ability of MSCs to differentiate into osteogenic and adipogenic lineages, by blocking the up-regulation of early lineage commitment regulators. Our data infer that a certain basal level of OCT4 is required to mediate these processes. Previous studies also showed that OCT4 expression is directly involved in the trans-differentiation of MSCs towards cardiomyocytes and neurons. Moreover, the potential role of OCT4 as a regulator of MSC multipotency is in agreement with previous reports demonstrating that the enhanced expression of this factor in neonatal tissue-derived MSCs is associated with an improved plasticity in comparison with their adult counterparts. Further studies are warranted to address the specific molecular mechanisms regulating OCT4 expression in MSCs and their response to differentiation signals.

## 4. Materials and Methods

### 4.1. Bone Marrow-Derived Mesenchymal Stromal Cells

All animal protocols were approved by the Favaloro University Animal Care and Use Committee (DCT0218). Bone marrow MSCs were collected from the femur and tibia of individual adult FVB mice. Ficoll density gradient was used to isolate viable mononuclear cells from BM samples (Ficoll-Paque PLUS, GE Healthcare–Amersham Biosciences). The mononuclear cell fraction was suspended in complete culture medium, composed of DMEM low glucose basal medium (Life technologies), supplemented with 10% fetal bovine serum (FBS) and 1% antibiotic–antimicotic solution (Life technologies). Cells were plated at 20 × 10^6^ cells/75 cm^2^ and incubated at 37 °C in a humidified incubator with 5% CO_2_. When the adherent layer reached 70–80% confluence, the cells were detached using 0.25% trypsin and 1 mM EDTA, and serially passaged at 4000 cells/cm^2^ every 5–7 days until fourth passage (P4) cells were obtained. Surface phenotype analysis and lineage differentiation assays were done to confirm that cells conform with minimal criteria defining MSCs [16]. Flow cytometry analysis was performed using the following antibodies: CD44 (APC-labelled, eBioscience), CD73 (PE-labelled, BD Pharmingen), CD90 (PE-labelled, Abcam), CD105 (PE-labelled, eBioscience), CD14 (PE-labelled, eBioscience), CD34 (PE-labelled, BD Pharmingen), and CD45 (APC-labelled, BD Pharmingen). For each marker, MSCs (2 × 10^4^ cells) were centrifuged, re-suspended in 100 μL of PBS, and stained by adding 2 μL of the corresponding antibody (1:50 dilution) for 30 min. After washing, fluorescence was evaluated in a Beckman Coulter Cytomics FC-500 and data analyzed using FlowJo software (TreeStar).

### 4.2. OCT4 Silencing

We used the mammalian expression plasmid vector pSilencer 4.1-CMV puro (Life Technologies) to produce a hairpin siRNA molecule for OCT4. The target sequence of siRNA for OCT4 was 5′-AGGAGCACGAGTGGAAAGCAA-3′. The construct was made by ligating complementary oligonucleotides that create the stem–loop structure of 21 bp into the BamHI and HindIII sites of the pSilencer 4.1-CMV vector. The construction was confirmed by DNA sequencing. The pSilencer 4.1-CMV control plasmid containing a scrambled siRNA was used as a negative control (scr siRNA, Life Technologies). Cells were transfected with the different constructs using Metafectene Easy+ (Biontex), according to the manufacturer’s instructions. Twenty-four hours post transfection, transfected cells were selected using puromycin (2.5 µg/mL).

### 4.3. Quantitative RT-PCR

Total RNA was extracted from cells using TRIzol reagent (Invitrogen) and one microgram RNA was reverse transcribed into cDNA using random primers (High-Capacity cDNA Reverse Transcription Kit, Applied Biosystems). Quantitative RT-PCR was performed by using specific primers to detect the expression of pluripotency factors and adipocyte- and osteoblast-specific differentiation genes (Supplementary Table S1). Samples were assayed using Power SYBR Green master mix on a Step-One real-time PCR system (Applied Biosystems) under standard cycling conditions, followed by a melting curve analysis. Specific amplification products were also confirmed by automatic DNA sequencing. The threshold cycle (Ct) values were normalized against the reference gene glyceraldehyde-3-phosphate dehydrogenase (GAPDH) and data are presented as the fold change in gene expression, relative to the untreated control [37].

### 4.4. Bisulfite Genomic Sequencing

Genomic DNA was purified from OCT4-silenced MSCs (siOCT4) and scrambled control siRNA transfected cells (siScr). Bisulfite conversion was performed using the MethylCode Bisulfite Conversion Kit (Invitrogen). A 533 bp region of the OCT4 promoter was amplified by nested PCR, with primers designed to recognize the bisulfite-converted DNA only [38] (Supplementary Table S1). PCR products were gel-purified with QIAquick Gel Extraction Kit (Quiagen) and then cloned into bacteria by pGEM-T easy cloning (Invitrogen). Sequencing data were analyzed using BISMA software [39]. Bisulphite conversion efficiency of non-CpG cytosines was higher than 95% for all individual clones for each sample.

### 4.5. Western Blotting

Protein extracts were prepared from purified nuclear fractions of MSCs. Briefly, cells were resuspended in lysis buffer containing 50 mM Tris–HCl (pH = 7.5), 1% Triton X-100, 137.5 mM NaCl, 10% Glycerol, 1 mM sodium vanadate, 1X protease inhibitors cocktail (Sigma). Lysates were incubated on ice for 15 min and centrifuged at 10,000 g for 15 min at 4 °C. Supernatant was collected (cytoplasmic fraction) and the nuclear pellet was separately resuspended in the same lysis buffer containing 0.2% SDS and briefly sonicated. Nuclear lysates were centrifuged at 10,000 g for 15 min at 4 °C and the supernatant was collected (nuclear fraction). Protein concentrations were determined using the micromethod of Bradford (Bio-Rad, CA). Nuclear samples (20 µg protein) were separated on a 10% SDS-PAGE gel and transferred to a HyBlot CL film (Denville Scientific, NJ, USA). Membranes were blocked using 1X PBST with 5% (w/v) non-fat dried milk for 1 h and hybridized overnight at 4 °C with an anti-OCT4 primary antibody (1:800, ab19857, Abcam). After washing with TBST buffer, membranes were incubated with an anti-rabbit HRP-conjugated secondary antibody and washed five times for 5 min with TBST buffer. Bound antibodies were visualized using ECL Plus Western Blotting detection system (GE Healthcare), according to the manufacturer’s instructions.

### 4.6. Osteogenic Differentiation

MSCs were seeded in 12-well plates at 1 × 10^4^ cells/cm^2^ and osteogenic differentiation was induced by DMEM, supplemented with 10% FBS, 10 mM β-glycerophosphate, 50 μM L-ascorbic acid-2-phosphate, and 0.1 μM dexamethasone. Medium was replaced three times weekly. After 21 days, Alizarin Red S staining and calcium mineral content quantitation were performed as described by Stanford et al. [40]. Briefly, cultures were fixed in ice-cold 70% ethanol for 1 h and then stained for 10 min with 40 mM Alizarin Red S solution (pH = 4.2) at room temperature. Cells were washed with PBS to reduce non-specific staining and photographed. A quantitative de-staining procedure was carried out using 10% (w/v) cetylpyridinium chloride in 10 mM sodium phosphate (pH = 7.0) for 15 min at room temperature. Alizarin Red S concentrations were determined by absorbance measurement at 570 nm, using a standard curve. For alkaline phosphatase (ALP) activity assays, cells were harvested at day 0, 7, and 14, and lysed in 0.05% Triton X-100. Samples were incubated at 37 °C for 30 min in 150 μL reaction buffer containing 1 M diethanolamine (pH = 10), 10 mM p-nitrophenyl phosphate (Sigma), and 1 mM MgCl_2_. The reactions were stopped by the addition of 5 μL of 1 N NaOH, and the absorbance was taken at 405 nm on a microplate reader (VersaMax, Molecular Devices). ALP activity was expressed in nmol/min µg of protein. Total protein concentration was determined using Bradford reagent (BioRad).

### 4.7. Adipogenic Differentiation

MSCs were seeded into 12-well plates at 2 × 10^4^ cells/cm^2^ and cultured to 80% confluency. For adipogenic differentiation, cells were incubated in DMEM high glucose containing 10% FBS, 0.5 mM methylisobutylxanthine, 10 μg/mL insulin, 1 μM dexamethasone, and 60 μM indomethacin. Adipogenic induction medium was replaced three times weekly. After 15 days, cells were washed twice with PBS and fixed with 4% formaldehyde for 30 min. Lipid droplets were stained with 0.5% Oil Red O in isopropanol/distilled water (60:40) for 30 min. Cells were washed twice with PBS and photographed under a microscope. The extent of differentiation was subsequently determined by dye extraction, using 4% Nonidet P40 in isopropyl alcohol, followed by spectrophotometry at 520 nm.

### 4.8. Statistical Analysis

Continuous variables are expressed as mean ± SD. The unpaired Student *t*-test was used to evaluate statistical significance between siScr and siOCT4 groups. One-way ANOVA with post hoc Tukey’s test was used to compare the expression of pluripotency genes between untreated MSCs, siScr, and siOCT4 groups. A value of *p* < 0.05 was considered statistically significant.

## Supplementary Materials

Supplementary materials can be found at https://www.mdpi.com/1422-0067/20/13/3268/s1.
