# Supplementary material for: OCT4 Silencing Triggers Its Epigenetic Repression and Impairs the Osteogenic and Adipogenic Differentiation of Mesenchymal Stromal Cells
Source: Int J Mol Sci. 2019 Jul 3;20(13):3268. doi: 10.3390/ijms20133268 (PMC6651015; doi:10.3390/ijms20133268)
Supplement: Supplementary file 1 [file ijms-20-03268-s001.pdf]

OCT4 silencing triggers its epigenetic repression and impairs the osteogenic and adipogenic differentiation of mesenchymal stromal cells

Ricardo Malvicini, Diego Santa-Cruz, Natalia Pacienza and Gustavo Yannarelli

Supplementary material

Supplementary Table 1. DNA primer sequences.

Primer	Sequence (5'-3')	Size (bp)
Gene expression		
OCT4-forward	CAATGCCGTGAAGTTGGAG	119
OCT4-reverse	CCAAGGTGATCCTCTTCTGC	
SOX2-forward	ACATGTGAGGGCTGGACTG	140
SOX2-reverse	CGTTTCGCTGCGGAGAT	
NANOG-forward	TCTGGGAACGCCTCATCA	128
NANOG-reverse	AGAGGCAGGTCTTCAGAGGAA	
ALP-forward[1]	AACCCAGACACAAGCATTCC	213
ALP-reverse[1]	CCAGCAAGAAGAAGCCTTTG	
RUNX2-forward[1]	CCACCACTCACTACCACACG	250
RUNX2-reverse[1]	TCAGCGTCAACACCATCATT	
COL1a1-forward[1]	CACCCTCAAGAGCCTGAGTC	250
COL1a1-reverse[1]	CGGGCTGATGTACCAGTTCT	
OCN-forward[1]	TTCTGCTCACTCTGCTGACC	251
OCN-reverse[1]	TTTGTAGGCGGTCTTCAAGC	
C/EBPα-forward[1]	GATAAAGCCAAACAACGCAAC	257
C/EBPα-reverse[1]	CTAGAGATCCAGCGACCCGA	
PPARγ2-forward[1]	TTTTCCGAAGAACCATCCGAT	343
PPARγ2-reverse[1]	ACAAATGGTGATTTGTCCGTT	
FABP4-forward[2]	ACACCGAGATTTCCTTCAAACTG	88
FABP4-reverse[2]	CCATCTAGGGTTATGATGCTCTTCA	
GAPDH-forward	ATGGTGAAGGTCGGTGTGA	81
GAPDH-reverse	CTCCACTTTGCCACTGCAA	
Bisulfite sequencing[3]		
OCT4-out forward	GAGGATTGGAGGTGTAATGGTTGTT	621
OCT4-out reverse	CTACTAACCCATCACCCCCACCTA	
OCT4-in forward	TGGGTTGAAATATTGGGTTTATTT	533
OCT4-in reverse	CTAAAACCAAATATCCAACCATA	

[1] Zhang *et al.*, J Biol Chem. 2008;283:4723-9.

[2] Jang M and Jung MH, Biochem Biophys Res Commun. 2015;456(1):80-5.

[3] Gao *et al.*, Cell Stem Cell 2013;12(4):453-69.
